# The effect of short term exposure to outdoor air pollution on fertility

**DOI:** 10.1186/s12958-021-00838-6

**Published:** 2021-10-06

**Authors:** Mireia González-Comadran, Bénédicte Jacquemin, Marta Cirach, Rafael Lafuente, Thomas Cole-Hunter, Mark Nieuwenhuijsen, Mario Brassesco, Buenaventura Coroleu, Miguel Angel Checa

**Affiliations:** 1 Department of Obstetrics and Gynecology, Hospital del Mar, Barcelona, Spain; 2 Barcelona Research Infertility Group, IMIM Institut Hospital del Mar d’Investigacions Mèdiques, Barcelona, Spain; 3 Univ Rennes 1, Inserm, EHESP, Irset (Institut de recherche en santé, environnement et travail) – UMR_S 1085, F-35000 Rennes, France; 4 Institute for Global Health (ISGlobal), Barcelona, Spain; 5 Department of Experimental and Health Sciences, Universitat Pompeu Fabra, Barcelona, Spain; 6 CIBER Epidemiología y Salud Pública (CIBERESP), Madrid, Spain; 7 Centro de Infertilidad y Reproducción Humana (CIRH), Barcelona, Spain; 8 Section of Environmental Health, Department of Public Health, University of Copenhagen, Copenhagen, Denmark; 9 Hospital Universitari Quiron Dexeus, Salud de la Mujer Dexeus, Barcelona, Spain; 10 Universidad Autónoma de Barcelona, Barcelona, Spain; 11 Fertty, ClÍnica de ReproducciÓn Asistida, Barcelona, Spain; 12 Reproductive Medicine Division at Hospital del Mar de Barcelona, Passeig Marítim 25-29, 08003 Barcelona, Spain

**Keywords:** Acute exposure, Particulate matter, Nitrogen dioxide, PM_2.5_, PM_10_, NO_2_, Fertility, Pregnancy, Miscarriage

## Abstract

**Background:**

There is evidence to suggest that long term exposure to air pollution could be associated with decreased levels of fertility, although there is controversy as to how short term exposure may compromise fertility in IVF patients and what windows of exposure during the IVF process patients could be most vulnerable.

**Methods:**

This prospective cohort study aimed to evaluate the impact of acute exposure that air pollution have on reproductive outcomes in different moments of the IVF process. Women undergoing IVF living in Barcelona were recruited. Individual air pollution exposures were modelled at their home address 15 and 3 days before embryo transfer (15D and 3D, respectively), the same day of transfer (D0), and 7 days after (D7). The pollutants modelled were: PM_2.5_ [particulate matter (PM) ≤2.5 μm], PM_coarse_ (PM between 2.5 and 10μm), PM_10_ (PM≤10 μm), PM_2.5_ abs, and NO_2_ and NOx. Outcomes were analyzed using multi-level regression models, with adjustment for co-pollutants and confouding factors. Two sensitivity analyses were performed. First, the model was adjusted for subacute exposure (received 15 days before ET). The second analysis was based on the first transfer performed on each patient aiming to exclude patients who failed previous transfers.

**Results:**

One hundred ninety-four women were recruited, contributing with data for 486 embryo transfers.

Acute and subacute exposure to PMs showed a tendency in increasing miscarriage rate and reducing clinical pregnancy rate, although results were not statistically significant. The first sensitivity analysis, showed a significant risk of miscarriage for PM_2.5_ exposure on 3D after adjusting for subacute exposure, and an increased risk of achieving no pregnancy for PM_2.5_, PM_coarse_ and PM_10_ on 3D.

The second sensitivity analysis showed a significant risk of miscarriage for PM_2.5_ exposure on 3D, and a significant risk of achieving no pregnancy for PM2.5, PM_coarse_ and PM10 particularly on 3D. No association was observed for nitrogen dioxides on reproductive outcomes.

**Conclusions:**

Exposure to particulate matter has a negative impact on reproductive outcomes in IVF patients. Subacute exposure seems to increase the harmful effect of the acute exposure on miscarriage and pregnancy rates. Nitrogen dioxides do not modify significantly the reproductive success.

## Introduction

Air quality has risen in interest worldwide over the past decades. According to the World Health Organization (WHO), approximately 92% of the world’s population [1] lives in areas where air quality exceeds their limits recommended for annual mean of particulate matter with a diameter of less than 2.5 micrometers (PM_2.5_) [2], affecting both developed and developing countries. Cities are often hotspots of air pollution, with Barcelona ranking among the top polluted cities in Europe.

In 2015, ambient PM_2.5_ was ranked as the fifth greatest mortality risk factor [3]. Between 4 and 9 million deaths were attributable to ambient air pollution, representing 7.6% of global deaths, and 103.1 million disability-adjusted life-years [3].

There is extensive evidence regarding the effect of air pollution on cerebrovascular [3–5] and ischemic heart disease [3, 6–9], chronic obstructive pulmonary disease [3, 10, 11], asthma [12, 13] other respiratory disorders [3, 5, 14, 15], and carcinogenicity [3, 16, 17], among others [18–20].

The effects of long-term exposure to air pollution on fertility [21–25] and perinatal outcomes [26–32] are also well documented. However, there is some controversy in the published literature regarding the effect of short-term exposure to air pollutants, and what phases during the reproductive process are women most vulnerable to this exposure. The heterogeneity in the study design, conducted among women undergoing IVF makes it more difficult to draw firm conclusions. Most published studies are retrospective, and there is little evidence from prospective trials. Some studies observed a decrease in pregnancy rate among women exposed to higher concentrations of nitrogen dioxide (NO_2_) [33, 34] and particulate matter 10 micrometers or less in diameter (PM_10_) [33] during different phases of the menstrual cycle during the IVF treatment. Other studies have described increases in miscarriage rate associated with higher exposures to PM_10_ [33, 35]. Gaskins et al., instead, used proximity to major roadways to measure the exposure to pollutants derived from traffic emissions and observed lower reproductive success in women living closer to traffic roads. In addition, there is uncertainty as to how each pollutant individually compromises fertility and what are the most relevant windows of exposure to be assessed.

The current study aims to assess the effect that short-term exposure to air pollution has on fertility, and what are the phases of the menstrual cycle that make women most vulnerable.

## Materials and methods

### Study design

A prospective observational cohort study was designed to evaluate the effect of short-term exposure to air pollutants on fertility, using the IVF model to accurately assess the different phases of the menstrual cycle. Participants were recruited between January 2014 and January 2018.

Our study population comprised women living in the area of Barcelona, who underwent one or more IVF cycles at Hospital del Mar de Barcelona, CIRH (*Centro de Infertilidad y Reproducción Humana*) and Hospital Universitari Quirón-Dexeus. Oocyte donation cycles and cancelled cycles were excluded from the study. Upon recruitment and prior to the embryo transfer (ET), a questionnaire assessing environmental exposure was delivered to each participant. Women that did not deliver the questionnaire were also excluded.

This study was supported by the public funding *Instituto de Salud Carlos III* (PI13/00454).

### Data collection

Exposure to environmental factors residential addresses as well as variables that have a detrimental effect of fertility were included in the questionnaire, such as smoking [36], demographic and socio-economic variables [37, 38], Body mass index (BMI) [39, 40], dietary habits [41], physical activity [42, 43] and lifestyle [44]. Regarding the dietary habits, a score was assigned to each participant based on data extracted from the questionnaire and following the validated brief Mediterranean diet screener (bMDS), which estimates the adherence to Mediterranean diet.

Data regarding the clinical diagnosis, and the IVF treatment as well as the reproductive outcomes were collected from clinical records. The reproductive outcome was measured by serum hCG test performed 10 days after the ET and the ultrasound performed 2 weeks after the hCG test and/or at any time during the first trimester. Women were classified based on their reproductive outcome: “no pregnancy”, “miscarriage” and “clinical pregnancy”. Miscarriage was defined as serum hCG level greater that 5mUI/ml, associated with a pregnancy loss occurring during the first trimester of pregnancy. Clinical pregnancy was determined by ultrasound examination during the first trimester.

### Exposure assessment to outdoor air pollution

For acute exposure, mean concentrations of PM_2.5_, PM_2.5_ abs, PM_10_, PM coarse, NO_2_ and NOx were modelled at home addresses of each participant in different windows during the implantation process: a) during the 3 days before ET (3D), beginning of the secretory phase; b) the same day of the ET (D0), during the window of implantation; and d) during the 7 days after (D7), once the implantation process has begun. To estimate individual subacute exposure we calculated the average daily exposure during the 15 days before the ET (15D), which corresponds to the proliferative phase of the menstrual cycle.

The exposure estimates were obtained from the ESCAPE study (European Study of Cohorts for Air Pollution Effects) that is described elsewhere [45]. Briefly, the spatiotemporal exposure assessment approach was based on land use regression modeling following a standardized protocol. Residential addresses were obtained and geocoded. We estimated the daily concentration according to the windows described above for each address for each transfer. The temporal adjustment factor based on routine monitoring data was done according to ESCAPE guidelines [46].

### Laboratory air quality

The IVF laboratory uses a centered high-efficiency particulate air (HEPA) system that supplies filtered air without particles to the IVF laboratory, as well as carbon filters to remove volatile organic compounds. Additionally, Coda® Inline® filters were placed between the carbon dioxide circuit and the incubators, and replaced every six months.

### Statistical analysis

As each participant could contribute with more than one ET, and assuming similarities between the ET from the same participants, fitting traditional regression models would lead to false inferences, hence multilevel regression models were required [47, 48]. Due to the characteristics of the outcome (categorical) a multilevel logistic regression analysis was performed. Results from this model showed the risk of “miscarriage” and “no pregnancy” compared with the outcome “clinical pregnancy”.

After an initial crude analysis to assess the effect of acute and subacute exposure to pollutants on reproductive outcomes, *a priori* we expressed all results relative to fixed increments in each pollutant, defined before analysis, 5 μg/m^3^ for PM_2.5_ and PM coarse, 10 μg/m^3^ for PM_10_ and PM_2.5_abs, and 20 μg/m^3^ for NO_2_ and NOx, as suggested by ESCAPE guidelines [46]. All analyses were performed using Stata software, version 13.1 (StataCorp).

Adjusted models were performed including known factors to have a detrimental effect on reproductive outcomes, variables that are associated with systemic inflammation and variables directly correlated with air pollution exposure. These confounders were age, BMI, physical activity, smoking intensity (measured in pack/day [49]), adherence to Mediterranean diet, socioeconomic status, number and quality of the embryos transferred, and the type of endometrial preparation (stimulated cycle versus natural or artificial cycle).

We performed two sensitivity analyses. First, the model for each pollutant was adjusted for the subacute exposure received during the proliferative phase, received during the 15 days before ET. The rationale behind this analysis is that some groups have observed a different response to the exposure of air pollutants among individuals with different basal levels of systemic inflammation [50, 51]. Hence the effect of air pollutants around the time of the ET could be influenced by cumulative effect of the exposure received during the proliferative endometrial phase, serving as a susceptibility factor.

Second, we analyzed the results including only the first transfer for each patient using classical logistic regression model, excluding patients who failed previous transfers for reasons that could bias our results and that were not controlled by the confounders included in the model.

Models evaluating the effect of particulate matter were adjusted for NO_2_, and models evaluating nitrogen oxides (NO_2_ and NOx) were adjusted for PM_2.5_. The goal was to assess the association between particulate matter and nitrogen oxides in the reproductive outcomes. Prior to this analysis, a potential correlation was addressed with the Person’s correlation coefficients, and high correlations were assumed for coefficients higher than 0.7.

We expressed all results relative to fixed increments in each pollutant, defined before analysis, 5 μg/m3 for PM_2.5_ and PM coarse, 10 μg/m3 for PM_10_ and PM_2.5_abs and 20 μg/m3 for NO_2_ and NOx. All analyses were fit using Stata software, version 13.1 (StataCorp).

### Ethics approval

Ethics approval was obtained (no. 2013/5249/I) from the Clinical Research Ethical Committee in Parc de Salut Mar, Barcelona, Spain.

## Results

One hundred ninety-four patients were included in the study, that contributed with 486 embryo transfers.

The mean age was 36.9 ± 4.02 years, mean BMI was 22.97 ± 3.69 kg/m^2^, and mean Anti-Müllerian Hormone (AMH) was 2.45 ± 2.62 ng/mL. 29.84% were fresh embryo transfers, and the mean number of embryos transferred per cycle was 1.56 ± 0.52. No differences were detected when comparing the mean number of embryos transferred in fresh versus frozen-thawed ET (p=0.61). The clinical pregnancy rate was 22.02% and clinical and biochemical miscarriage rates were 9.26 and 10.91%, respectively.

An analysis of the patient's characteristics according to the results from the IVF cycles was performed. The number of MII oocytes retrieved and the number of fertilized oocytes were significantly lower among women who did not achieve a pregnancy as compared with women in the clinical pregnancy and miscarriage groups (*p* = 0.029 and 0.0198, respectively). Results are described in Table 1.

**Table 1:** Patient’s characteristics and IVF parameters

	Clinical pregnancy	No pregnancy	Miscarriage	p*
	Mean ± standard deviation / Median (p25, p75) / n (%)			
Maternal age, years	36.44 ± 0.33	37.20 ± 0.25	36.39 ± 0.44	0.109
BMI	22.60 (0.36)	22.91 (0.22)	23.62 (0.41)	0.120
Normal weight (18-24.9 kg/m2)	65 (69.15)	177 (79.02)	69 (74.19)	0.187
Overweight (25-29.9 kg/m2)	16 (17.02)	33 (14.73)	16 (17.20)	
Obesity (≥ 30 kg/m2)	3 (3.19)	7 (3.13)	5 (5.38)	
Active smokers (%)	16 (14.29)	42 (15.50)	17 (16.50)	0.990
Smoking status (pack/day)	0.30 (0.20, 0.55)	0.40 (0.25, 0.60)	0.50 (0.30, 0.50)	0.880
Low socio-economic status	43 (41.35)	114 (46.34)	58 (53.19)	0.246
Sedentarism	76 (68.47)	208 (76.75)	73 (70.87)	0.332
Mediterranean diet (score)	14.5 (15, 20)	17 (15, 19)	17 (16, 20)	0.294
Ovarian stimulation				
Agonist (%)	19 (16.96)	51 (18.82)	16 (15.53)	0.738
Antagonist (%)	93 (83.04)	220 (81.18)	87 (84.47)	
Duration of stimulation, days	11 (9, 12)	11 (10, 12)	11 (10, 12)	0.786
No. MII Oocytes retrieved	8 (6, 11)	7 (4, 11)	9 (5,14)	0.029
No. Embryos fertilized	5 (3, 7)	4 (3, 7)	5 (4, 8)	0.019
Fresh embryo transfers (%)	39 (34.82)	80 (29.52)	26 (25.24)	0.304
No. Embryos transferred	1.59 ± 0.51	1.52 ± 0.52	1.64 ± 0.52	0.092
Blastocyst transfer (%)	30 (26.79)	31 (11.44)	23 (22.33)	<0.001

*p25* 25th percentile, *p75* 75^th^ percentile, *n* number, *%* percentage, *BMI* Body Mass Index (expressed as kilograms / meter^2^), *MII* Metaphase II oocytes

The statistical analysis was performed using the χ2 test for categorical variables (expressed as n (%)), with one-way variance ANOVA for numerical variables with normal distribution (expressed as Mean ± Standard deviation), and Kruskall Wallis test for numerical variables with non-normal distribution (expressed as Median (p25, p75))

* *p* values ≤ 0.05 are considered statistically significant

During the study period, daily mean concentrations were modelled at their home addresses on concrete periods during the menstrual cycle (15D, 3D, D0, D7 and annual average), as registered on Table 2. For PM_2.5_ and PM_10_, average concentrations exceeded the levels recommended by the WHO Air quality guidelines [35], and for NO_2_, the average concentration was close to the upper limit of the permitted exceedances (10 μg/m3, 20 μg/m3 and 40 μg/m3 respectively) for subacute and acute exposures [52].

**Table 2:** Exposure to air pollutants around the time of the embryo transfer

	15D	3D	D0	D7
NO_2_ (μg/m3)	36.30 ± 15.38	36.05 ± 19.03	38.01 ± 21.90	36.62 ± 17.63
NO_x_ (μg/m3)	62.13 ± 32.29	62.09 ± 41.44	65.41 ± 46.17	62.74 ± 36.90
PM_2.5_ (μg/m3)	10.42 ± 3.51	10.35 ± 4.62	10.37 ± 5.04	10.57 ± 4.19
PM_2.5_ abs (1 unitat)	1.92 ± 0.82	1.62 ± 1.12	2.02 ± 1.29	1.93 ± 0.96
PM coarse (μg/m3)	12.17 ± 4.13	12.14 ± 5.40	12.16 ± 6.07	12.43 ± 4.97
PM_10_ (μg/m3)	21.87 ± 6.52	21.83 ± 9.13	21.90 ± 10.41	22.31 ± 8.19

*NO*_*2*_ nitrogen dioxide, *NOx* nitrogen oxide, *PM*_*2.5*_ particulate matter (PM) with aerodynamic diameter ≤2.5 μm, *PM*_*2.5*_ *abs* a surrogate of black carbon, *PM coarse* PM with aerodynamic diameter between 2.5 and 10 μm, *PM*_*10*_ PM with aerodynamic diameter ≤ 10 μm, *15D* period of 15 days before the embryo transfer, *3D* period of 3 days before the embryo transfer, *D0* the day of the embryo transfer, *D7* period of 7 days after the embryo transfer

Values are expressed as means ± standard deviation, values for PM_2.5absorbance_ expressed in 10^-5^*m^-1^ m

The correlation analysis performed to test potential collinearity between pollutants, showed low correlation between pollutants, except for PM_2.5_abs and NO_2_, which appeared highly correlated (*p* > 0.80)  (Table 3). These results rule out the possibility of adjusting the effect of PM_2.5_abs by NO_2_ exposure.

**Table 3:** Correlation between particulate matter and nitrogen oxides

Pollutant		NO_2_			
	Exposure window	15D	3D	D0	D7
PM_2.5_	15D	0.48			
	3D		0.56		
	D0			0.51	
	D7				0.45
PM_2.5_ abs	15D	0.88^a^			
	3D		0.90^a^		
	D0			0.91^a^	
	D7				0.91^a^
PM coarse	15D	0.55			
	3D		0.56		
	D0			0.56	
	D7				0.59
PM_10_	15D	0.53			
	3D		0.55		
	D0			0.55	
	D7				0.59
		PM_2.5_			
NO_2_	15D	0.55			
	3D		0.56		
	D0			0.53	
	D7				0.60
NOx	15D	0.57			
	3D		0.59		
	D0			0.58	
	D7				0.59

*NO*_*2*_ nitrogen dioxide, *PM*_*2.5*_ particulate matter (PM) with aerodynamic diameter ≤2.5 μm, *PM*_*2.5*_ *abs* a surrogate of black carbon, *PM coarse* PM with aerodynamic diameter between 2.5 and 10 μm, *PM*_*10*_ PM with aerodynamic diameter ≤ 10 μm, *NOx* nitrogen oxide, *15D* period of 15 days before the embryo transfer, *3D* period of 3 days before the embryo transfer, *D0* the day of the embryo transfer, *D7* period of 7 days after the embryo transfer

^a^High correlations indicate collinearity, and result in unstable estimates when adjusting models by co-pollutants

Acute and subacute exposure to particulate matter show a tendency in increasing miscarriage rate and reducing clinical pregnancy rate, although results are not statistically significant (data not shown).

**Fig. 1:**
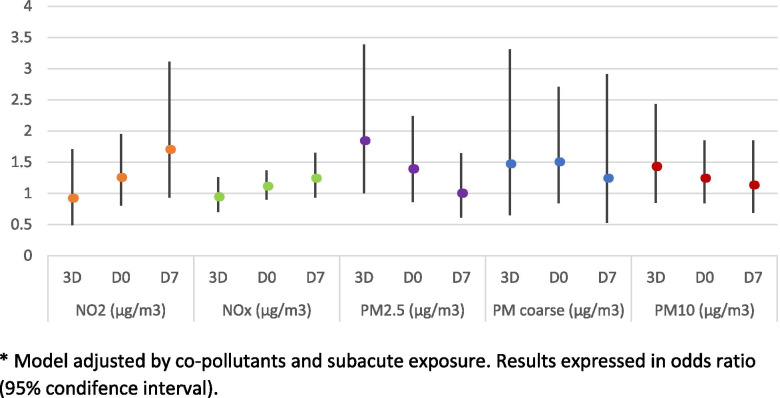
Risk of miscarriage compared with the risk of clinical pregnancy for the acute exposure (from 3 days prior to ET to 7 days after the ET). * Model adjusted by co-pollutants and subacute exposure. Results expressed in odds ratio (95% condifence interval)

**Fig. 2:**
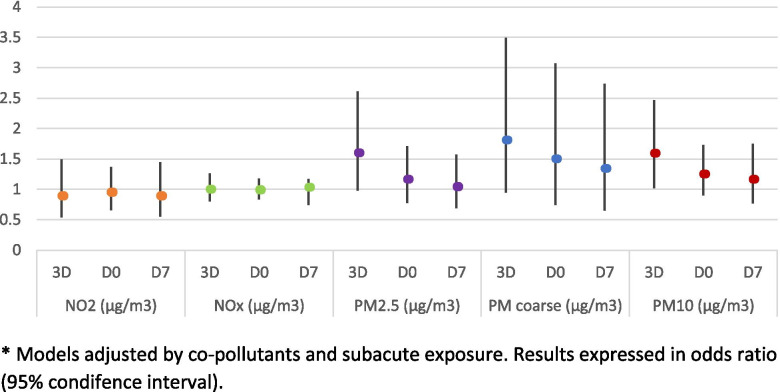
Risk of achieving no pregnancy compared with the risk of clinical pregnancy for the acute exposure (from 3 days prior to ET to 7 days after the ET). * Models adjusted by co-pollutants and subacute exposure. Results expressed in odds ratio (95% condifence interval)

In the first sensitivity analysis, an increased risk of miscarriage was observed compared with the clinical pregnancy rate during 3D and D0 for all PMs, reaching statistical significance for the exposure to PM_2.5_ three days before the embryo transfer (OR 1.84, 95% CI 1.00 – 3.39), as observed in Fig. 1. The risk of achieving no pregnancy was also increased for particulate matter, being significant or close to significance for exposures during 3D for PM_2.5_ (OD 1.60, 95% CI 0.98 – 2.61), PM coarse (OR 1.81, 95% CI 0.95 – 3.49) and PM_10_ (OR 1.59, 95% CI 1.02 – 2.47) (Fig. 2).

Neither the acute exposure to NO_2_ nor NO_x_ showed significant associations with the risk of miscarriage or the risk of not achieving a pregnancy for any of the key days (Figs. 1 and 2).

In the second sensitivity analysis, that was based on the first embryo transfer cycle of each participant, a clear tendency on the reproductive outcomes is observed for particulate matter. With regard to the risk of miscarriage, a significant association is observed for PM_2.5_ during 3D (OR 3.80, 95% CI 1.13 – 12.80). The risk of achieving no pregnancy was also significantly increased for PM_2.5_ during 3D (OR 2.72, 95% CI 1.20 – 6.18), for PM coarse during 3D and D0 (OR 3.38, 95% CI 1.04 – 10.92, and OR 2.37, 95% CI 1.06 – 5.30, respectively). For PM_10_, results were close to significance during 3D and D0 (OR 1.87, 95% CI 0.97 – 3.58 and OR 1.55, 95% CI 0.99 – 2.41, respectively). No effect was observed for NO_2_ or NOx on the reproductive outcomes when adjusting for PM_2.5_.

## Structured discussion/comment

### Principal findings

Particulate matter could affect fertility. Particularly short-term exposure during the onset of the secretory phase and at the time of the embryo implantation could have a detrimental effect on the endometrium reducing clinical pregnancy rate and increasing miscarriage rates. In addition, the cumulative exposure to air pollution received during the proliferative phase could confer vulnerability to increases of acute exposure.

### Results

The results of this study show that acute increases in the exposure to particulate matter, particularly PM_2.5_ and PM_10_ during the onset of the secretory phase and around the time of the embryo implantation, have a negative effect on reproductive outcomes. An increase in the risk of miscarriage was observed at higher exposure to PMs, results that were only significant for PM_2.5_ at the onset of the secretory phase. Similarly, a decrease in pregnancy rate was also observed for PMs, particularly at the onset of the secretory phase and around the time of the embryo implantation, being significant or close to significance for PM_2.5_ and PM_10_ (Figs. 1 and 2). While, NO_2_ does not seem to have any significant effect on reproductive outcomes. The statistical analysis was performed adjusting for co-pollutants (particulate matter models were adjusted for NO_2_, and nitrogen oxide models were adjusted for PM_2.5_).

In a first sensitivity analysis, we explored the effect of subacute and acute exposures on reproductive outcomes, and whether this subacute exposure during the proliferative phase could interfere with the effect that acute exposure has on fertility around the time of the embryo implantation. Results from this analysis showed a global increase in the risks of both miscarriage and “no pregnancy” with respect to clinical pregnancy (Figs. 1 and 2, respectively) compared with that of the crude model and the one adjusted only for covariates (data not shown). These findings suggest that the cumulative exposure received during the proliferative phase of the endometrial cycle could serve as a susceptibility factor, modifying the effect of the exposure received around the time of the implantation.

There is epidemiological evidence to suggest that long-term exposure to air pollution has a deleterious effect on reproductive life [21–23] and perinatal outcomes [26–32]. There is also some evidence among women undergoing IVF to suggest that short-term exposure could impair reproductive outcomes [23]. Results from this study support the findings described in previous retrospective trials that have addressed this issue [21, 22, 33–35, 53]. The literature published is highly heterogeneous in terms of both the target population evaluated and the exposure periods analyzed, and although most authors evaluated exposures ranging from the early follicular development to the hCG test after embryo transfer [33–35, 53, 54], results reported are also conflicting.

### Clinical implications

Women seeking for pregnancy who live in highly polluted cities could be advised to adapt their lifestyle to limit their exposure. Among women undergoing IVF, clinicians could consider to avoid ETs when peaks of air pollution are expected, recommend these women to go out for fresh air away from cities before the procedure, or even be advice to not naturally ventilate the home or vehicles, and instead use air conditioning when possible.

Air pollution is a major public health concern because of its ubiquity and known negative effect on health, and it has been identified as a worldwide health priority. However, global but also local public health measures are needed to reduce pollution and prevent these related harmful effects, in particular their effect on reproductive health, through public health policies in the field of pollution regulation, and limitations on traffic and fuel type, among others.

### Research implications

The present study provides some insight on several aspects that have not been addressed yet in the previous studies, such as the effect that the cumulative exposure to air pollutants during the proliferative phase of the menstrual cycle has on fertility, and how this can modify the effect that acute exposure has on reproductive success.

This effect has been described by some authors [50, 51], who reported a significantly higher effect of pollutants on markers of inflammation among individuals with elevated markers of basal inflammation. Indeed, an increase in the systemic inflammatory response during the proliferative phase could confer vulnerability by modifying the activation or suppression of genes in the endometrium during this phase, thereby modify the effect that acute exposure could have during the implantation process.

However, this concept of susceptible individuals needs to be explored in further prospective trials. In this regard, it is essential to identify subgroups with greater vulnerability to the harmful effect of pollutants, especially those under the effect of systemic inflammation. Of particular relevance, aspects related to lifestyle such as obesity, metabolic syndrome, sedentary lifestyle, and smoking, among others.

### Strengths and limitations

This study has some limitations to consider; most importantly the sample size. In environmental epidemiology, there are many confounding factors, and estimates tend to be low hence the sample size needs to be large to be able to detect significant differences, even though our power calculation gave us power above 80% for PM_2.5_. Besides, there is potential bias in air pollution exposure model determinants, as well as selection bias of the population included.

Nevertheless, this study has strengths that need to be promoted. Analyzing the effect of pollutants among IVF women provided an opportunity to assess what moments during the menstrual cycle patients are most vulnerable, and gain insight into the mechanisms by which air pollutants impair fertility. Besides, the prospective nature of the study allows an accurate control of confounders associated to either reproductive success, systemic inflammation or exposure to pollutants. Moreover, models were adjusted for co-pollutants, allowing us to detect a greater effect of PM exposure when controlling for NO_2_. Finally, the hierarchical structure of the study population allowed a greater representativeness of what happens in clinical practice, increasing the external validity of the results in the general population seeking pregnancy.

## Conclusions

This study shows that acute exposure to particulate matter has a negative effect on fertility particularly during the onset of the secretory phase and at the time of the embryo implantation, while exposure during the proliferative phase seems to increase the harmful effect of the acute exposure. NO_2_ does not seem to have any effect.

## Data Availability

The datasets generated and/or analysed during the current study are not publicly available due the extension and complexity of the analysis, but are available from the corresponding author on reasonable request

## Abbreviations

IVF: In vitro fertilization
ET: Embryo transfer
PM_2.5_: Particulate matter (PM) ≤2.5 μm in diameter
PM_coarse_: PM between 2.5 and 10μm
PM_10_: PM≤10 μm
PM_2.5_ abs: PM _2.5_ absorvance refers to the soot content of PM_2.5_
NOx: Nitrogen oxides
NO_2_: Nitrogen dioxide
15D: 15 days before the embryo transfer
3D: 3 days before the embryo transfer
D0: The same day of the embryo transfer
D7: 7 days after the embryo transfer
ESCAPE: European Study of Cohorts for Air Pollution Effects
LUR: Land use regression
HEPA: High-efficiency particulate Air system
VOCs: Volatile organic compounds
WHO: World Health Organization
bMDS: Brief Mediterranean diet screener
hCG: Human chorionic gonadotropin
CIRH: Centro de Infertilidad y Reproducción Humana
BMI: Body mass index
AMH: Anti-mullerian hormone
MII: Metaphase II oocytes
OR: Odds ratio
CI: Confidence interval
